# Sepsis in Critically Ill Patients: Epidemiology, Risk Factors, and Role of Matrix-Assisted Laser Desorption/Ionization-Time of Flight Mass Spectrometry for Identification of Sepsis-Causing Organisms

**DOI:** 10.7759/cureus.22445

**Published:** 2022-02-21

**Authors:** Kuldeep Singh Sindhlian, Zia Arshad, Prashant Gupta, Vipin K Singh, Gyan Prakash Singh, Haider Abbas

**Affiliations:** 1 Department of Anaesthesiology, King George’s Medical University, Lucknow, IND; 2 Department of Anaesthesia and Critical Care, King George’s Medical University, Lucknow, IND; 3 Department of Mircrobiology, King George’s Medical University, Lucknow, IND; 4 Department of Emergency Medicine, King George’s Medical University, Lucknow, IND

**Keywords:** antibiotics, sepsis-causing organism, maldi-tof ms, intensive care unit, sepsis

## Abstract

Background

Matrix-assisted laser desorption/ionization-time of flight mass spectrometry (MALDI-TOF MS) is a rapid mass spectrometry technology for species identification. It is a useful, fast, and accurate tool for routine laboratory analysis. This study aimed to investigate the epidemiology of sepsis-causing organisms in patients admitted to tertiary-level intensive care units (ICU), the role of MALDI-TOF MS in species identification, and patients’ clinical outcomes.

Methodology

A prospective observational study was performed in a tertiary-level ICU for one year. The first blood, urine, and endotracheal (ET) aspirate samples were sent before starting antibiotics. We received the antibiotic sensitivity report within 48 hours or earlier using MALDI-TOF MS. Treatment was modified based on MALDI-TOF MS reports. Response to treatment was monitored, and clinical outcomes were noted based on ICU stay. Patients were followed up until discharge, shifting to referring parent unit, or death.

Results

This study included 200 patients admitted to ICUs who at the time of admission did not have a fever. The most common organisms were *Acinetobacter baumannii, Klebsiella pneumonia, *and *Escherichia coli* in ET aspirates; *Candida albicans* and *Enterococcus faecium *in urine; and *Pseudomonas aeruginosa, K. pneumonia*, and *A. baumanii*in blood. Of the 200 patients, 130 (65%) shifted to the parent unit ward, and 70 (35%) patients died, with an ICU stay of 12.89 ± 6.51 days. There was no significant difference in mortality when organisms grew from either ET or urine compared with sterile samples. If organisms resistant to all primary antibiotics grew from ET, mortality was 60.6%. Mortality was 56.8% if isolates were in the blood.

Conclusions

Early MALDI-TOF MS-based species identification and appropriate antibiotics initiation play a key role in the treatment and care for critically ill patients with sepsis. MALDI-TOF MS has the potential to significantly aid sepsis management.

## Introduction

Matrix-assisted laser desorption/ionization-time of flight mass spectrometry (MALDI-TOF MS) is a rapid mass spectrometry technology developed in the late 1980s. It is a useful, fast, and accurate tool for routine laboratory analysis. Several studies have reported that the introduction of MALDI-TOF MS reduces intensive care unit (ICU) and hospital stays of patients with bacteremia and/or candidemia and markedly decreases hospital costs [1,2]. However, to our knowledge, the impact of MALDI-TOF MS on the clinical outcomes of patients with sepsis, especially critically ill patients requiring admission to an ICU, remains poorly understood. MALDI-TOF MS utilization in a clinical microbiology laboratory has markedly increased over the past 10 years [3]. Over time, platforms have progressively advanced, with significant improvements in the software, interpretive rules, and databases. Consequently, there is limited value in comparing results for any category of organisms using a retrospective literature review. In this study, we hypothesized that rapid MALDI-TOF MS-based identification on top of a well-established antimicrobial stewardship program (ASP) would significantly improve antibiotic management compared with an ASP using conventional identification even in a hospital setting with a low prevalence of resistant organisms. Consequently, we conducted this prospective study of a one-year duration investigating the epidemiology, risk factors, and role of MALDI-TOF MS in species identification of sepsis-causing organisms.

This study aimed to investigate the epidemiology of sepsis-causing organisms in patients admitted to tertiary-level ICU care, the role of MALDI-TOF MS in species identification, and patients’ clinical outcomes.

## Materials and methods

Study setting and design

This prospective observational study was conducted in a tertiary-level ICU from September 2019 to August 2020. In this study, we performed convenience non-probability sampling. The sample size was determined using the following equation: N = (r + 1/r) x σ^2^(Zβ + Zα/2)^2^/(difference)^2^, where n is the number needed in each group, r is the ratio of control to cases (valued at 1, an equal number of cases and control), σ is the standard deviation from the reference study, Zβ is the standard normal variate for a set power (0.84 for 80% power), Zα/2 is the standard normal variate for the level of significance (1.96 for 95% confidence interval, CI), and the difference of means in the reference study (d = 0.7) was 196.85. Finally, 200 cases included in the study. The minimum sample size for this study was statistically calculated based on the study by Chatterjee et al. [4].

Inclusion and exclusion criteria

We included patients aged 16-60 years who were admitted to the ICU, those with new-onset fever after 48 hours of hospital admission, and those with new-onset neutropenia with fever. Because the study was conducted in adult ICU, pediatrics patients were excluded. As the number of patients above the age of 60 years was low, we included patients between the age group of 16 and 60 years.

We excluded patients unwilling to provide consent, those having fever at admission, and those developing fever within 48 hours of admission. Additionally, we excluded all patients who presented with the diagnosis of sepsis at the time of admission.

Study procedure

The study was performed in a tertiary-level ICU. If a patient met the inclusion criteria and was willing to participate, informed consent was obtained. The first sample was collected at the time of admission. Repeat samples after developing fever with two sets of peripheral blood cultures were collected, each containing at least 8-10 mL. One sample from the central venous line was also collected (if applicable) along with peripheral blood culture. We received an antibiotic sensitivity report within 48 hours or earlier using MALDI-TOF MS. Treatment was initiated based on MALDI-TOF MS and susceptibility test findings. Response to treatment was monitored, and clinical outcomes were documented based on the length of ICU stay. The cases were followed up till the patients were discharged, shifted to a referring parent unit ward, or deceased.

Ethical approval

Ethical approval was obtained from the Institutional Ethics Committee (King George’s Medical University, Lucknow, India; DCGI registration number: ECR/262/inst/up/2013/RR-19), with approval number 102 ECM II B-THESIS/P79.

Statistical analysis

The data were entered in Microsoft Excel and analyzed using SPSS version 23 (IBM Corp., Armonk, NY USA). The Student’s t-test was used to analyze parametric data, and the Mann-Whitney U test was used to analyze non-parametric data. Fisher’s test was used to analyze categorical data. The confidence interval (CI) was set at 95%, the alpha value was 0.05, the power of 1-ß was 0.8, and the level of statistical significance was set at P-values of <0.05.

## Results

In this study, the most common isolated organisms were *Acinetobacter baumannii, Klebsiella pneumoniae, Escherichia coli*, and *Pseudomonas aeruginosa* in endotracheal (ET) aspirates; *Candida albicans*,* Enterococcus faecium*,* and C. glabrata in urine*;* and P. aeruginosa*,* A. baumannii*,* and K. pneumoniae* in the blood(Table 1)*. *This study was conducted in a government-aided hospital where we received referred patients with sepsis-induced multiorgan dysfunction syndrome (MODS) with multidrug-resistant infections. Cross-infection may be one of the causes for the high incidence of multidrug-resistant organisms. Because we only included patients who developed a fever after admission, it may be one of the causes of high incidence.

**Table 1:** Distribution of organisms from various samples. ETA: endotracheal aspirate

	Site (N = 200)	Organism	Numbers (N)
Sample 1	ETA (N = 186)	Acinetobacter baumannii	91
		Klebsiella pneumoniae	14
		Escherichia coli	9
		Pseudomonas aeruginosa	8
		Proteus mirabilis	3
		Staphylococcus aureus	3
		Sterile	58
	Non-intubated patients (n = 14)		14
Sample 2	Urine (N = 194)	Candida albicans	10
		Enterococcus faecium	9
		Candida glabrata	6
		Enterococcus faecalis	6
		Escherichia coli	3
		Candida auris	3
		Klebsiella pneumoniae	3
		Candida orthopsilosis	3
		Trichosporon asahii	3
		Sterile	137
	Sample not sent (N = 6)		6
Sample 3	Blood (N = 197)	Acinetobacter baumannii	9
		Pseudomonas aeruginosa	10
		Klesbiella pneumoniae	9
		Stenotrophomonas maltophilia	3
		Enterococcus faecalis	4
		Staphylococcus aureus	2
		Sterile	160
	Sample not sent (N = 3)		3
Miscellaneous	Drain (N = 2)	Escherichia coli	2
	Placenta membrane (N = 3)	Escherichia coli	3
	Pus (N = 10)	Escherichia coli	6
		Klebsiella pneumoniae	3
		Pseudomonas aeruginosa	2
		Staphylococcus epidermidis	2
	Sputum (N = 2)	Acinetobacter baumannii	2

**Figure 1:**
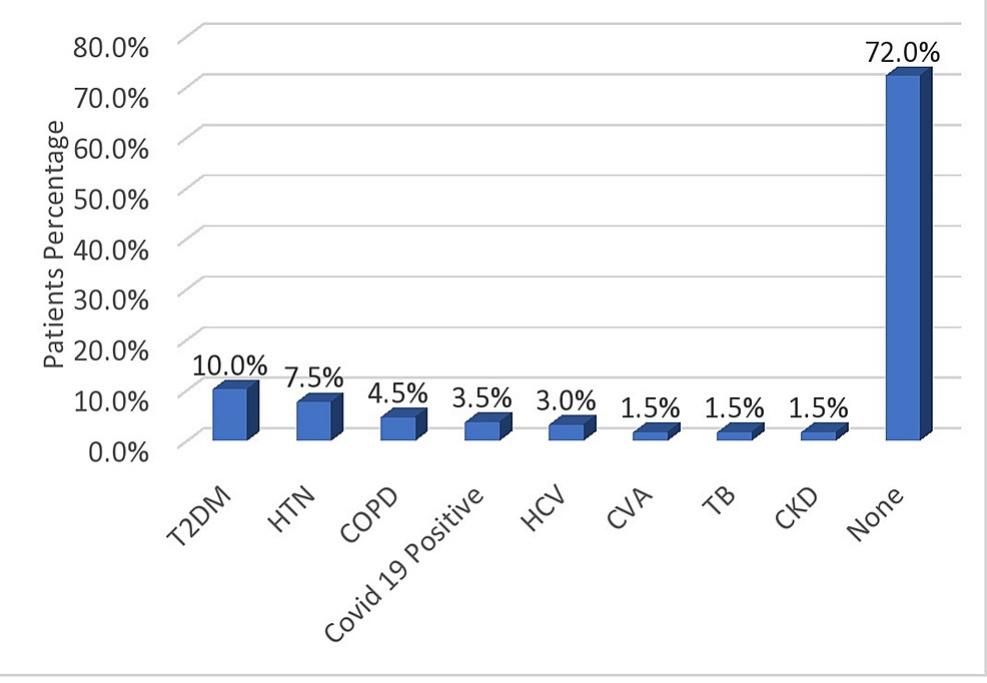
Comorbidities seen in the study population. T2DM: type 2 diabetes mellitus; HTN: hypertension; COPD: chronic obstructive pulmonary disease; COVID-19: coronavirus disease 2019; HCV: hepatitis C virus; CVA: cerebrovascular accident; TB: tuberculosis; CKD: chronic kidney disease

In the study, 10% of the studied patients had diabetes, followed by hypertension (7.5%), chronic obstructive pulmonary disease (4.5%), severe acute respiratory syndrome coronavirus 2 (SARS-CoV-2) infection (coronavirus disease 2019, COVID-19) (3.5%), and hepatitis C virus (HCV) infection (3.0%), whereas 72.0% did not have any of the above risk factors (Figure 1).

In 2018, mortality was 35.36% (325/919), and in 2019, it was 35.34% (328/928). Thus, in our study, mortality was approximately 35%. Data were collected from the institutional records maintained in the ICU (Table 2).

**Table 2:** Year-wise mortality.

Year	Total patients	Mortality	Percentage
2018	919	325	35.36%
2019	928	328	35.34%
2020 (present study)	200	70	35.0%

Based on the antibiotics susceptibility tests of organisms isolated from ET aspirates, *A. baumanii *was most susceptible to tetracycline, tobramycin, levofloxacin, and meropenem. In blood, *P. aeuroginosa* was the most common isolated organism and was most susceptible to piperacillin/tazobactam and gentamycin. In urine, *Candida* spp. was susceptible to both fluconazole and voriconazole (Tables 3, 4). Patients who developed symptoms or neutropenia were treated with fluconazole.

**Table 3:** Antibiotic susceptibility pattern of the organisms in ET and miscellaneous causing septicemia. R to all: resistant to all primary antibiotics; S to all: sensitive to all primary antibiotics, Piptaz: piperacillin/tazobactam; T/S: trimethoprim/sulfamethoxazole; ET: endotracheal

Site (N = 200)	Organism	Numbers (N)	R to all	Gentamycin	Amikacin	Levoflox	Tetracycline	Tobramycin	Piptaz	Polymixin	S to all	Imipenem	Meropenem	Tigecycline	T/S	Amoxycillion	Aztreonam	Cefoxitin
ET (N = 186)	Acinetobacter baumannii	91	33	5	4	10	35	12	3	3	5	3	3	2	3	----	---	
	Klebsiella pneumoniae	14	3		3		5					3			3	3	3	
	Escherichia coli	9						3	3			3	9		3		3	
	Pseudomonas aeruginosa	8			6		2	3	3			2					2	
	Proteus mirabilis	3										3	3					
	Staphylococcus aureus	3			3		3								3			
	Sterile	58	--	--	--	--	--	--	--	--	--	--	--	--	--	--	--	
Drain (N = 2)	Escherichia coli	2		2	2													2
Placental membrane (N = 3)	Escherichia coli	3					3										3	
Pus (N = 7)	Klebsiella pneumoniae	3			3		3											
	Pseudomonas aeruginosa	2									2							
	Staphylococcus epidermidis	2									2							
Sputum (N = 2)	Acinetobacter baumannii	2					2	2										

**Table 4:** Antibiotic susceptibility pattern of organisms in urine causing septicemia. S to all: sensitive to all primary antibiotics; T/S: trimethoprim/sulfamethoxazole

Site	Organism	No.	Fluconazole	Voriconazole	T /S	S to all	Nitrofurantoin	Aztreonam	Tetracycline	Teicoplanin	Norfloxacin	Ciproflox	Norflox	Linezolid	Vancomycin	Tobramycin	Amikacin
Urine (N = 194)	Candida albicans	10	10	10													
	Enterococcus faecium	9							3			3	3	3	3		
	Candida glabrata	6	6	6													
	Enterococcus faecalis	6		3			3		3	3	3						
	Candida tropicalis	8	8	8													
	Escherichia coli	6			3	3											
	Candida auris	3	3	3													
	Klebsiella pneumoniae	3						3	3								
	Candida orthopsilosis	3	3	3												3	3
	Trichosporon asahii	3				3											
	Sterile	137															
Pus (N = 3)	Escherichia coli	3						3									
Sample not sent (N = 3)	---	---															

Of a total of 200 patients, 130 (65%) were shifted to the parent unit, and 70 (35%) patients died, with an ICU stay of 12.89 ± 6.51 days (Table 5).

**Table 5:** Clinical outcomes of ICU patients. ICU: intensive care unit

Variable	Number of patients (N = 200)	Number of days in ICU
Shifted to the referring parent unit	130 (65.0)	13.73 ± 6.94
Expired	70 (35.0)	11.32 ± 5.29
Total		12.89 ± 6.51

Of a total of 200 patients, 186 patients were mechanically ventilated, and 37% of patients died, while the rest of the 14 patients were transferred to the referring parent unit (Table 6).

**Table 6:** Clinical outcomes in mechanically ventilated patients. ICU: intensive care unit

Variable	Number of patients (N = 186)	Number of days in ICU
Shifted to the parent unit	116 (62.4)	13.93 ± 6.95
Expired	70 (37.6)	11.32 ± 5.29
Total	186	12.95 ± 6.49

There was no significant difference in mortality when microorganisms grew either in ET aspirates or urine compared with sterile samples (insignificant P-value), but if microorganisms grew in blood, mortality was as high as 56.8% compared with 30.6% (Table 7).

**Table 7:** Mortality ratio for positive growth and sterile organisms on the basis of samples. ET: endotracheal

Organism	ET (N = 186)		Urine (N = 194)		Blood (N = 197)	
	Number	Mortality	Number	Mortality	Number	Mortality
Positive growth	128	50 (39.0%)	57	20 (35.1%)	37	21 (56.8%)
Sterile	58	20 (34.4%)	137	50 (36.5%)	160	49 (30.6%)
P-value	0.550		0.851		0.002	

If microorganisms growing in ET aspirates were resistant to all primary antibiotics, mortality was 60.6% (Table 8).

**Table 8:** Mortality in multidrug-resistant organisms in ETA. ETA: endotracheal aspirate

Site	Organism	Sensitivity	Mortality
ETA (N = 33)	Acinetobacter baumannii	Resistant to all antibiotics	20 (60.6%)

## Discussion

Sepsis is defined as life-threatening organ dysfunction caused by a dysregulated host response to infection [5]. The Surviving Sepsis Campaign recommends attempting to prove the presence of an infection by methods such as microbiological testing [6]. The gold standard to detect bloodstream infections is blood culture in a liquid media [7], followed by cultural and biochemical identification of the culprit pathogen. Thus, rapid blood culture testing with pathogen identification is essential to establish a diagnosis and enable efficient therapy.

Severe sepsis and septic shock are the most common causes of morbidity and mortality in critically ill patients. Angus et al. [7] reported severe sepsis incidence of 2.26 cases per 100 hospital discharges, with 51.1% requiring intensive care. The overall mortality rate was 28.6%, which increased in patients with comorbidities. The overall mortality in gram-negative bacteremia is 25%. When septic shock develops, the mortality increases to 50-60% [8]. However, despite dramatic improvements in our knowledge of the pathogenesis, diagnosis, and therapeutic and supportive care, the mortality in septic patients remains unacceptably high, ranging from 30% to 50% in severe sepsis, and increasing to 50-87% in septic shock patients [9].

In the study, we found that the most common organism isolated from ET aspirates (n = 186) was* A. baumanii *(91), followed by *K. pneumoniae* (14) and *E. coli *(9). In urine, out of 197 samples, the most common organisms were *C. albicans *(10) and *E. faecium* (9). In blood (n = 197), the most common organisms were * P. aeuroginosa* (10), *K. pneumoniae* (9), and *A. baumanii* (9). This study was conducted in a government-aided hospital where we received referred patients with sepsis-induced MODS with multidrug-resistant infections. Cross-infection can be a cause for the high incidence of multidrug-resistant organisms. As we included patients who developed a fever after admission that may be one of the causes of high incidence. Our results are consistent with the increasing trend of gram-negative septicemia in ICU [8]. Thus, there is a need for early diagnosis and species identification to initiate species-specific antibiotics and early shifting from empirical to a specific therapy. Traditional techniques for culture and antibiotic susceptibility usually take four to five days to provide results and delay the initiation of appropriate antibiotics. Our findings were comparable to the study by Khwannimit and Bhurayanontachai [9] who reported that the respiratory tract was the most common site for both community and hospital-acquired infections.

In our study, 10% of patients had diabetes, followed by hypertension (7.5%), chronic obstructive pulmonary disorder (4.5%), COVID-19 (3.5%), and HCV (3.0%), whereas 72% did not have any of the above risk factors. Our findings were consistent with those of Zhong et al. [10] who reported diabetes mellitus, chronic obstructive pulmonary disease, and chronic hepatic insufficiency as the major risk factors for bacterial bloodstream infections. About 72% of our patients did not have any comorbidity; therefore, the data are inconclusive to draw results.

A major advantage of using MALDI-TOF MS was that it reduced the time between blood drawing and receiving the results about organisms causing sepsis [11]. Our findings were consistent with the previous study proving the efficacy of MALDI-TOF MS in critically ill patients [3]. We collected hospital data from our ICU to see any mortality benefit. In 2018, mortality was 35.36% (325/919), and in 2019, it was 35.34% (328/928). Thus, in our study, it was about 35%, although mortality is multifactorial and does not depend on one factor. Hence, we cannot conclude that MALDI-TOF MS has mortality benefits in sepsis patients, but it has an established role in early detection, early initiation of specific antibiotics, early de-escalation of antibiotics, and reduced ICU stay.

Considering the antibiotics susceptibility of the organisms isolated from ET aspirates, *A. baumanii* was most susceptible to tetracycline, tobramycin, levofloxacin, and meropenem. In blood*, P. aeuroginosa *was the most common isolated organism and was susceptible to piperacillin-tazobactam and gentamycin. In urine, isolated *Candida* spp. was susceptible to both fluconazole and voriconazole. Patients who developed symptoms or neutropenia were treated with fluconazole.

We observed clinical outcomes of the patients regarding ICU stay. Of a total of 200 patients, 130 (65%) patients shifted to the referring parent unit, and 70 (35%) patients died, with an ICU stay of 12.89 ± 6.51 days. There was no significant difference in mortality when microorganisms grew either in ET or urine compared with sterile samples. If microorganisms resistant to all primary antibiotics grew from ET, mortality was 60.6%. Mortality was 56.8% if the isolates were in the blood. Out of 200 patients, 186 patients were mechanically ventilated, with mortality of 37%, and the rest 14 patients were transferred to the parent referring unit. Therefore, mechanical ventilation is an independent risk factor of mortality in ICU. Zhong et al. [10] reported that in 117 patients, the mean ICU stay was 14 days. Further 28-day mortality was 35.0%, whereas 60-day mortality was 39.3%, and in-hospital mortality was 42.7%. Su et al. [1] also reported on the clinical impact of patients with bloodstream infection with different groups of viridians streptococci by using MALDI-TOF MS, concluding that the mean ICU stay was significantly higher in patients who survived than those who died (P < 0.01).

The present study has some limitations. Because it was a single-center study of one ICU, antibiogram and organisms may not reflect the actual burden. Moreover, there was no data to compare the results of MALDI-TOF MS with the traditional methods. Due to the SARS-CoV-2 infection, results became incomparable to the past data. The sample size was small to demonstrate any mortality benefit of MALDI-TOF MS and the inability to discriminate between related species. Additionally, MALDI-TOF MS is currently unable to differentiate E. coli from *Shigella*.

## Conclusions

As the incidence of gram-negative organisms is rapidly increasing in sepsis patients, early recognition and species identification can be achieved by MALDI-TOF MS, and initiation of appropriate antibiotics plays an important role in the treatment and care of patients. MALDI-TOF MS has the potential to revolutionize sepsis management. A major advantage of using MALDI-TOF MS was that it reduced the time between blood drawing and receiving the results regarding organisms causing sepsis. Although the time required for the identification by MALDI-TOF MS is 10 minutes/strain, we received the final sensitivity reports within 48 hours, which earlier used to take four to five days. MALDI-TOF MS is quick and accurate, providing species identification within two to six hours from positive growth to sensitivity testing and reporting. Our findings show that the use of MALDI-TOF MS has the potential to replace traditional methods of species identification, and routine use may have mortality benefits in patients with sepsis as sepsis management is time-specific. The tool has a role in the early initiation of specific therapy and early de-escalation of treatment. A larger randomized control trial is needed to establish mortality benefits.

## References

[1] Dellinger RP, Levy MM, Carlet JM (2008). Surviving Sepsis Campaign: international guidelines for management of severe sepsis and septic shock: 2008. Crit Care Med.

[2] Washington JA 2nd, Ilstrup DM (1986). Blood cultures: issues and controversies. Rev Infect Dis.

[3] Schubert S, Kostrzewa M (2017). MALDI-TOF MS in the microbiology laboratory: current trends. Curr Issues Mol Biol.

[4] Chatterjee S, Bhattacharya M, Todi SK (2017). Epidemiology of adult-population sepsis in India: a single center 5 year experience. Indian J Crit Care Med.

[5] Napolitano LM (2018). Sepsis 2018: definitions and guideline changes. Surg Infect (Larchmt).

[6] Kumar A, Roberts D, Wood KE (2006). Duration of hypotension before initiation of effective antimicrobial therapy is the critical determinant of survival in human septic shock. Crit Care Med.

[7] Angus DC, Linde-Zwirble WT, Lidicker J, Clermont G, Carcillo J, Pinsky MR (2001). Epidemiology of severe sepsis in the United States: analysis of incidence, outcome, and associated costs of care. Crit Care Med.

[8] Vincent JL, Sakr Y, Sprung CL (2006). Sepsis in European intensive care units: results of the SOAP study. Crit Care Med.

[9] Khwannimit B, Bhurayanontachai R (2009). The epidemiology of, and risk factors for, mortality from severe sepsis and septic shock in a tertiary-care university hospital setting. Epidemiol Infect.

[10] Zhong L, Zhang S, Tang K (2020). Clinical characteristics, risk factors and outcomes of mixed Candida albicans/bacterial bloodstream infections. BMC Infect Dis.

[11] Homolová R, Bogdanová K, Bardoň J, Kolář M (2020). [Direct identification of bacteria in blood cultures by MALDI-TOF MS]. Klin Mikrobiol Infekc Lek.

